# Ultra-thin ion exchange film on the ceramic supporter for output power improvement of reverse electrodialysis

**DOI:** 10.1038/s41598-019-54002-7

**Published:** 2019-11-25

**Authors:** Dong Hyeon Jung, Eui Don Han, Byeong Hee Kim, Young Ho Seo

**Affiliations:** Advanced Mechanical Engineering, Kangwon National University, 1 Gangwondaehak-gil, Chuncheon, 24341 South Korea

**Keywords:** Mechanical engineering, Applied physics

## Abstract

In this study, ultra-thin ion exchange film on the ceramic supporter (UTFCS) composed of thin polymer layer and nanoporous ceramic layer with low electrical resistance was developed. The electrical properties and permselectivity of UTFCSs were evaluated and the properties of UTFCSs were compared with other ion exchange membranes. Fabricated UTFCSs were applied to a reverse electrodialysis (RED) system to evaluate the output characteristics and compared with other ion exchange membranes. The power density of RED using UTFCS was 36.6 mW/m^2^, which was 8% higher than that of a commercial anion exchange membrane. In addition, possibility as power source was experimentally verified by driving LEDs. The proposed UTFCS can be applied not only to RED but also to energy development such as fuel cells and microbial cells.

## Introduction

**Figure 1:**
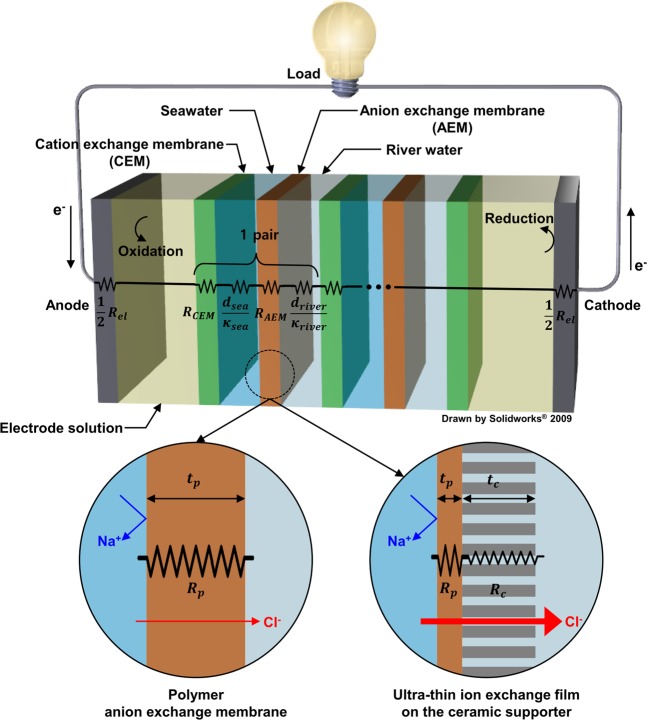
Illustration of reverse electrodialysis (RED) power generation system. Internal resistance of RED cell is sum of resistance of ion exchange membranes and solution parts. To reduce the internal resistance, ultra-thin ion exchange film on the ceramic supporter (UTFCS) was developed. UTFCS consists of ultra-thin polymer layer and ceramic supporter with nanoporous structure.

Salinity gradient energy (SGE) refers to the chemical energy present between solutions with different salinity concentrations, and the conversion of the salinity energy into electrical energy is referred to as salinity generation. Figure 1 shows a schematic diagram of reverse electrodialysis (RED) power generation system, which is a salinity generation technology. As shown in the figure, when the cation exchange membrane and the anion exchange membrane are alternately arranged and seawater and fresh water are flowed alternately, the diffusion of the ions occurs owing to the concentration gradient, thereby forming a potential at both the ends of the membrane. The potential causes redox reactions at the oxidation and reduction electrodes. Electricity is generated by inducing the movement of the electrons^1,2^. Determining the output of the RED power generation is the internal resistance of the RED stack and is expressed as follows^3^,

1 $${R}_{stack}=\frac{N}{A}({R}_{CEM}+{R}_{AEM}+\frac{{d}_{sea}}{{\kappa }_{sea}}+\frac{{d}_{river}}{{\kappa }_{river}})+{R}_{el}$$

where ${R}_{stack}$ is the internal resistance of the stack (Ω); *N* is the number of pairs of ion exchange membranes (-); *A* is the area of the ion exchange membrane (cm2); ${R}_{CEM}$ and ${R}_{AEM}$ are the area resistances of the cation and anion exchange membrane (Ω∙cm^2^), respectively; ${d}_{sea}$ and ${d}_{river}$ are the thickness of seawater and river water (cm), respectively; ${\kappa }_{sea}$ and ${\kappa }_{river}$ are the conductivity of seawater and river water (S/cm), respectively, and ${R}_{el}$ is the electrode resistance (Ω). Geise *et al*. reported that approximately 46% of the stack internal resistance is contributed by the resistance of the ion exchange membranes^4^. Lacey stated that low electrical resistance, high ion selectivity, mechanical strength, and durability of the ion exchange membranes are required to improve the output of RED^5^. Commercial ion exchange membranes sold by various manufacturers have high ion selectivity; however, because of their relatively high electrical resistance, research is required to reduce the electrical resistance of ion exchange membranes^6–9^. In order to reduce the electrical resistance of the ion exchange membrane, studies have been conducted to prepare an ion exchange membrane by adding a nanomaterial to the polymer^10^. Yang *et al*. showed that the conductivity of the ion exchange membrane increased three times by mixing aluminium oxide (Al_2_O_3_) nanoparticles with a polyvinyl alcohol(PVA)-based ion exchange membrane, and a 0.2–0.4 Ω∙cm^2^ area resistance was achieved^11^. Siracusano *et al*. evaluated zirconium oxide (ZrO_2_) in Nafion, and the sheet resistance of the ion exchange membrane was reported to reduce by 0.02 Ω∙cm^2^ ^12,13^. In addition, PVA has been mixed with sulfonated multi-walled carbon nanotubes or mixed with silicon dioxide to improve the electrical properties^14,15^. However, the nanomaterials are expensive, and carbon nanotubes have difficulty in being uniformly distributed in the polymer^16^. In addition to the method of mixing nanomaterials, research has been conducted to reduce the electrical resistance by changing the surface shape of the ion exchange membrane. The diffusion boundary layer around the ion exchange membrane hinders the movement of the ions and increases the electrical resistance. Pawlowski *et al*. used casting to fabricate ion exchange membranes with V-shaped patterns and columns, reducing the electrical resistance of the ion exchange membrane by 5 Ω∙cm^2^ ^17^. Lee *et al*. reduced the electrical resistance of the ion exchange membrane by 3.3 Ω∙cm^2^ by applying an alternating electric field to the ion exchange membrane solution to align the polymer chain in a certain direction^18^. However, a large amount of equipment is required. In addition to the above-mentioned methods, prior studies have evaluated a reduction of resistance through the chemical modification of the ion exchange membrane^19^, and the improvement of the electrical characteristics through mixing of a specific polymer^20^; however, most studies have focused on the mixing of nanomaterials and chemical modification. In addition, there is insufficient research on reducing the electrical resistance by reducing the thickness of the ion exchange membrane. Thin ion exchange membranes have a low mechanical strength and thus are not durable. Therefore, some studies prepared an ion exchange membrane by mixing inert polymers, such as polyvinylidene fluoride (PVDF) and polyacrylonitrile (PAN); however, this also has a thickness limit on the order of several tens of micrometres. When the mixing ratio of the inert polymer is high, the electrical resistance of the ion exchange membrane increases^20,21^. The above-mentioned studies all have disadvantages, such as a low mechanical strength and low chemical stability because they are made of polymers. Therefore, a thin ion exchange membrane with a low electrical resistance and excellent mechanical strength and chemical stability should be developed. In this study, a ultra-thin ion exchange film on the ceramic supporter (UTFCS) with a low electrical resistance, mechanical stiffness, and chemical resistance was proposed. Figure 1 shows the difference between the polymer anion exchange membrane and the UTFCS. The resistance of the polymer layer and the area resistance of the ceramic supporter can be expressed by the following equations.

2 $${R}_{p}={\rho }_{p}\cdot {t}_{p}$$

3 $${R}_{c}={\rho }_{c}\cdot {t}_{c}$$

Here, ${R}_{p}$ and ${R}_{c}$ are the area resistance (Ω∙cm^2^); ${\rho }_{p}$ and ${\rho }_{c}\,$are the specific resistance (Ω∙cm), ${t}_{p}\,$and ${t}_{c}$ are the thickness (cm) of the polymer and ceramic supporters, respectively. As shown in the figure, because the UTFCS has a thinner polymer layer than that of a general polymer anion exchange membrane, the area resistance of the polymer layer is low. A ceramic supporter with a porous structure capable of smoothly transferring ions serves as a support for the polymer layer and imparts mechanical strength and chemical stability. In this study, UTFCSs were fabricated, and the electrical resistance and ion selectivity were evaluated. The UTFCSs were applied to RED to evaluate and compare the power characteristics.

## Results and Discussion

### Electrical resistance and permselectivity of a UTFCS

**Figure 2:**
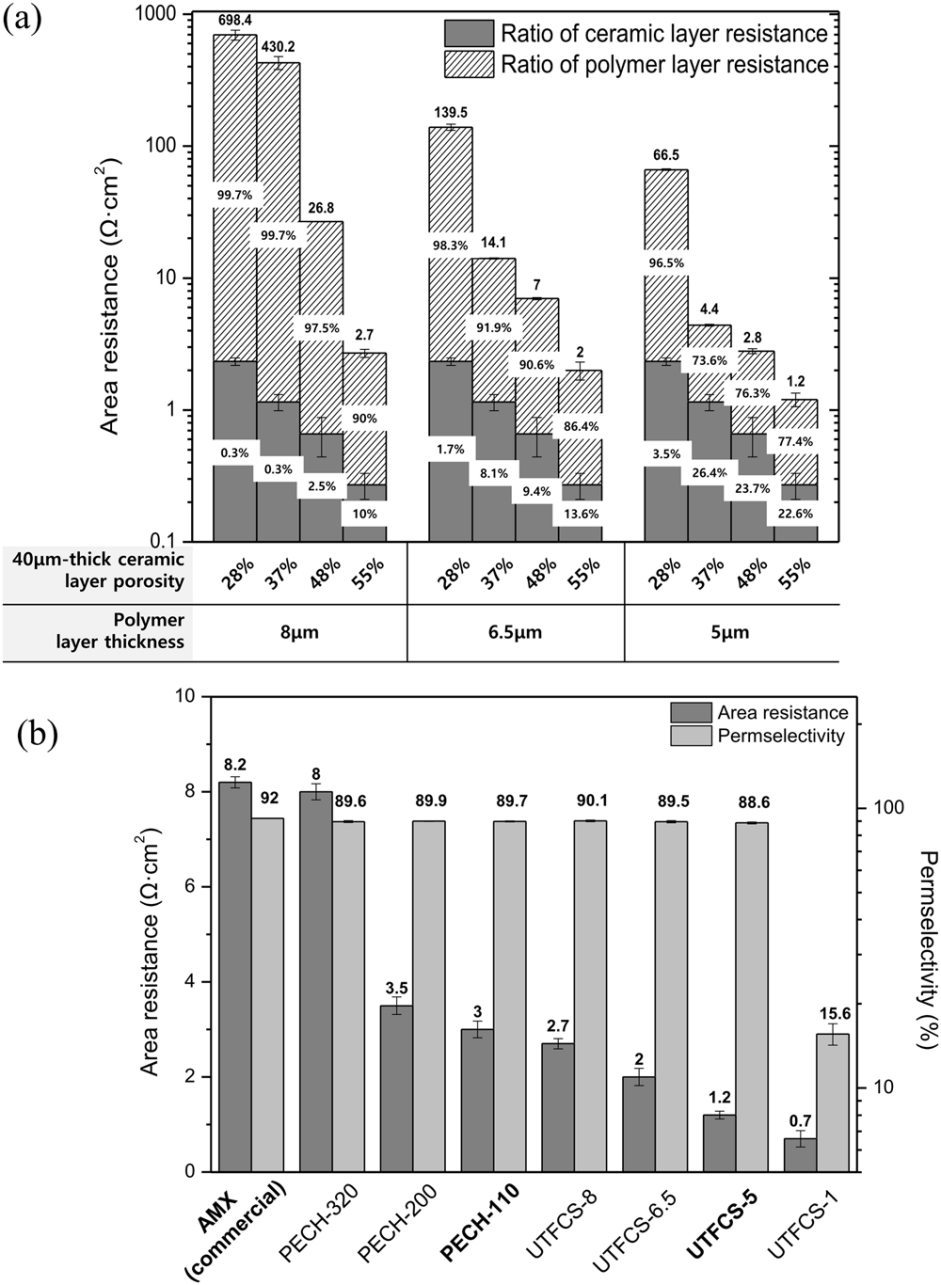
Electrical characteristics of ion exchange membranes, (**a**) Area resistance of ultra-thin ion exchange film on the ceramic supporter (UTFCS) depending on the thickness of polymer layer and the porosity of ceramic supporter. (**b**) Area resistance and permselectivity of commercial anion exchange membrane (AMX), polyepichlorohydrin (PECH) anion exchange membrane (PECH-320, PECH-200, PECH-110) and UTFCSs (UTFCS-8, UTFCS-6.5, UTFCS-5, UTFCS-1).

Figure 2(a) shows a graph of the area resistance according to the thickness of the polymer layer and the porosity of the ceramic supporter. The area resistance of the UTFCS decreased as the thickness of the polymer layer decreased and as the porosity of the ceramic supporter increased. The decreasing area resistance owing to the decrease of the polymer layer thickness can be explained by the shortening of the ion pathway through the polymer layer^10^. The decrease of the sheet resistance with an increasing porosity could be because the resistance of the pores is inversely proportional to the square of the pore diameter^22^. Meanwhile, when thickness of polymer layer is the same, ratio of the electrical resistance of polymer layer varies depending on the porosity of ceramic supporter. These results imply that the electrical resistance of the polymer layer is affected by the porosity of the ceramic supporter. This is believed to be the result of the combination of polymer layer with homogenous phase and ceramic supporter with heterogenous phase^23,24^. As porosity of ceramic supporter increases, the ratio of electrical resistance of polymer layer decreases. It can be seen from Eqs. (2) and (3) that the use of a ceramic supporter having a high porosity is advantageous in reducing electrical resistance. Therefore, in this study, a ceramic supporter with a porosity of 55% was used. In order to compare the properties of the UTFCS with other anion exchange membranes, various thicknesses of PECH polymer anion exchange membranes were fabricated. Figure 2(b) shows a graph of the area resistance and permselectivity of each anion exchange membrane. The beginning of the name refers to the material, and the end of the name refers to the thickness of the polymer layer. AMX, a commercial anion exchange membrane (ASTOM), was included as a comparative group. In this Figure, the area resistance decreased as the thickness of the PECH polymer anion exchange membrane decreased, and PECH-110 showed the lowest area resistance of 3 Ω∙cm^2^. In the UTFCS, UTFCS-1 showed the lowest sheet resistance of 0.7 Ω∙cm^2^. For the permselectivity, the PECH polymer anion exchange membrane showed no significant difference based on the thickness, whereas the UTFCS showed a slight decrease in the permselectivity as the polymer layer thickness decreased. UTFCS-1 showed a low permselectivity of 15%. UTFCS-5, which has a high ion selectivity of 88% and a sheet resistance of 1.2 Ω∙cm^2^, was applied to RED power generation. The output characteristics were compared with PECH-110 and AMX.

### Output power of RED using a UTFCS

**Figure 3:**
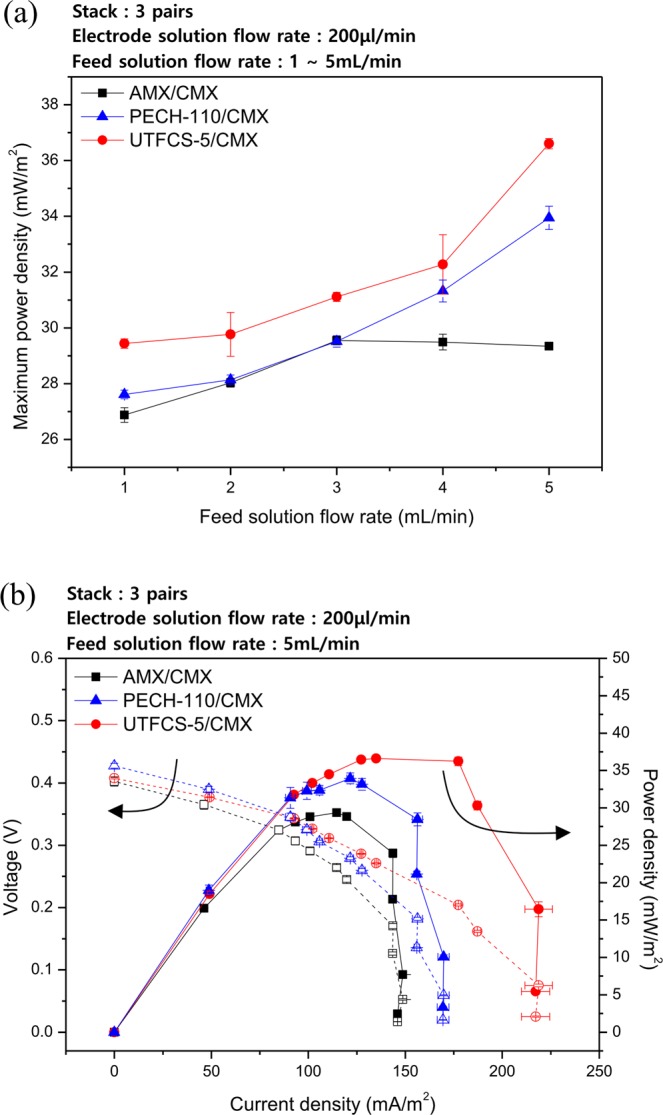
Output characterization of AMX, PECH-110, UTFCS-5, (**a**) maximum power density of each anion exchange membrane depending on the flow rate of feed solution, (**b**) I-V polarization curve and power density curve when the flow rate of feed solution is 5 mL/min.

The output characteristics of RED power generation using UTFCS-5 were evaluated and compared with those of AMX and PECH-110. The cation exchange membrane used for power generation was CMX, a commercial cation exchange membrane (ASTOM). The output characteristics of the RED power generation system were evaluated by setting the number of pairs of ion exchange membranes to three pairs and fixing the flow rate of the electrode solution to 200 μL/min. Seawater and river water, called as feed solution, were supplied at the same flow rate. Figure 3(a) shows a graph of the maximum power density of each anion exchange membrane based on the feed solution flow rate. At a flow rate of 1 to 5 mL/min, AMX exhibited a maximum power density of 29.5 mW/m^2^ at a flow rate of 3 mL/min. PECH-110 and UTFCS-5 exhibited a maximum power density of 33.9 mW/m^2^ and 36.6 mW/m^2^ at flow rate of 5 mL/min, respectively. Figure 3(b) shows a graph of the current-voltage curves and power density curves at the feed solution flow rate (seawater and river water flow rate) of 5 mL/min. When the UTFCS was used, the output was 8% higher than that with PECH-110 and 24% higher than that with AMX. As shown in Fig. 3(a), PECH-110 and UTFCS-5 tend to increase maximum power density as the feed solution flow rate increases, but AMX tends to decrease. The reason of the decrease of the power density of AMX/CMX cells at high flow rate is related with the increase of the internal resistance. Internal resistance in RED cells is typically changed by the distribution of ions in the feed solution. When RED cells are operated, the concentration polarization is developed at the membrane-solution interface due to differences of ion transport number between solution phase and the polymer membrane. This concentration polarization increases internal ohmic resistance^25,26^, but the concentration polarization is easily reduced by the flow of the feed solution which provides the efficient mixing of the fluid. However, the high flow rate of the feed solution increases the internal resistance of the river water compartment. In summary, there is an optimal flow rate of the feed solution, because the power density of typical RED cells is gradually increased and then decreased as the feed solution flow rate increases. These phenomena have been confirmed in several previous studies^25–28^. As shown in Fig. 3(a), the flow rate of 3 mL/min may be optimal in the commercial anion exchange membrane of AMX. On the other hand, the optimal flow rate of PECH-110 and UTFCS-5 will be above 5 mL/min. In short, both PECH-110 and UTFCS-5 more efficiently transported anions to river water side rather than AMX.

### LED operation test using the UTFCSs

**Figure 4:**
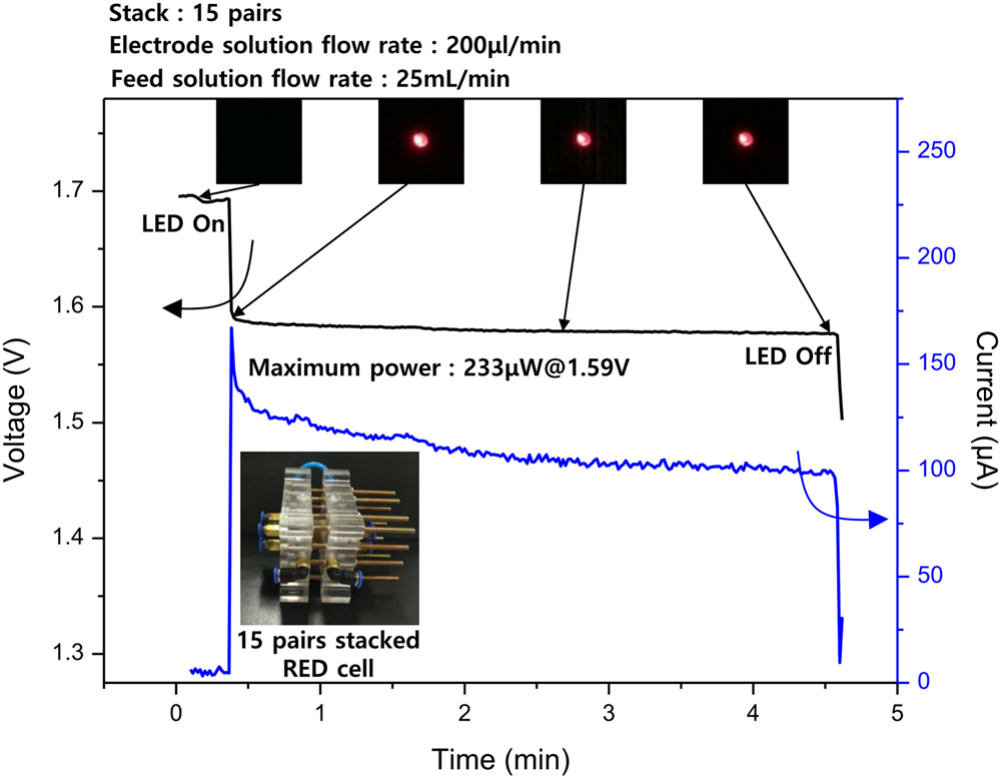
LED operation test using 15 pairs of ultra-thin ion exchange film on the ceramic supporter (UTFCS) and CMX cation exchange membrane. Maximum power is 233 μW at 1.59 V. LED is operated for about 4 minutes.

To verify the possibility as power source, an LED operation test was carried out using the 15 pairs of UTFCS-5/CMX and AMX/CMX. In the case of AMX/CMX cells could not operate LED due to low power density. Figure 4 shows the voltage and current data when the LED was operated by UTFCS/CMX cells. When the flow rate of the electrode solution was 200 μL/min and the flow rate of the feed solution was 25 mL/min, an open circuit voltage of approximately 1.7 V was obtained. When the load (LED) was applied, the LED was driven with a maximum power of approximately 233 μW at a voltage of 1.59 V, and for approximately 4 min. Therefore, the UTFCS fabricated in this study could be used as a power source.

## Conclusion

In this study, a UTFCS with a low electrical resistance and excellent mechanical strength and chemical resistance was proposed. In the polymer layer, a PECH-based anion exchange membrane was used to avoid the use of carcinogenic materials. The ceramic supporter was made of nanoporous alumina prepared through aluminium anodization. A UTFCS was fabricated by spreading the PECH-based anion exchange membrane solution onto the nanoporous alumina fabricated through the aluminium anodization and etching processes. In order to investigate the effect of the polymer layer thickness and porosity of the ceramic supporter on the electrical resistance of the UTFCS s, polymer layers with an 8 μm, 6.5 μm, and 5 μm thickness and ceramic supporters with a porosity of 28%, 37%, 48%, and 55% were fabricated. As the porosity of the ceramic supporter increased, the ratio of the area resistance of the polymer layer decreased. UTFCS s with high permselectivity of approximately 88% and a low area resistance of 1.2 Ω·cm^2^ were fabricated. The UTFCS s were applied to RED power generation to evaluate the output characteristics. The power density was 36.6 mW/m^2^, which was 8% higher than that of PECH-110 and 24% higher than that of AMX, a commercial anion exchange membrane. In addition, possibility as power source was experimentally verified by driving LEDs using 15 pairs of UTFCS-5 and CMX.

## Methods

### Ceramic supporter

An aluminium anodization process was performed to fabricate a ceramic supporter with a nanoporous structure. Nanoporous alumina is formed when a voltage is applied after immersing aluminium in an electrolyte solution with an anode and platinum as a cathode. In this study, high purity (99.999%) aluminium was anodized from 0.3 M phosphoric acid (Daejung chem) electrolyte, which can form a large pore diameter of 200~400 nm. The thickness of the ceramic supporter was controlled by deriving the sum of the charges applied through current monitoring during the aluminium anodization process. After the aluminium anodizing process, the unnecessary aluminium was removed using hydrochloric acid etchant. A barrier layer of nanoporous alumina was removed with a 0.1 M phosphoric acid etchant to form through-hole nanoporous alumina.

### Anion exchange membrane solution

Chloromethylation, one of the processes for the production of anion exchange membranes, is highly hazardous because it requires the use of chloromethyl methyl ether, a highly toxic carcinogen. Polyepichlorohydrin (PECH) contains a chloromethyl group; therefore, the chloromethylation process can be omitted. This study adopted the PECH-based anion exchange membrane fabrication method proposed by Guler *et al*.^21^. Here, 1 g of PECH (Sigma-Aldrich), 3 g of PAN (Sigma-Aldrich), and 8 g of 1,4-diazabicyclo [2.2.2] octane (DABCO, Sigma-Aldrich) were mixed with 28 g of dimethyl sulfoxide (DMSO, Sigma-Aldrich) and stirred at 80 °C for 9 h to prepare an anion exchange membrane solution.

### UTFCS

**Figure 5:**
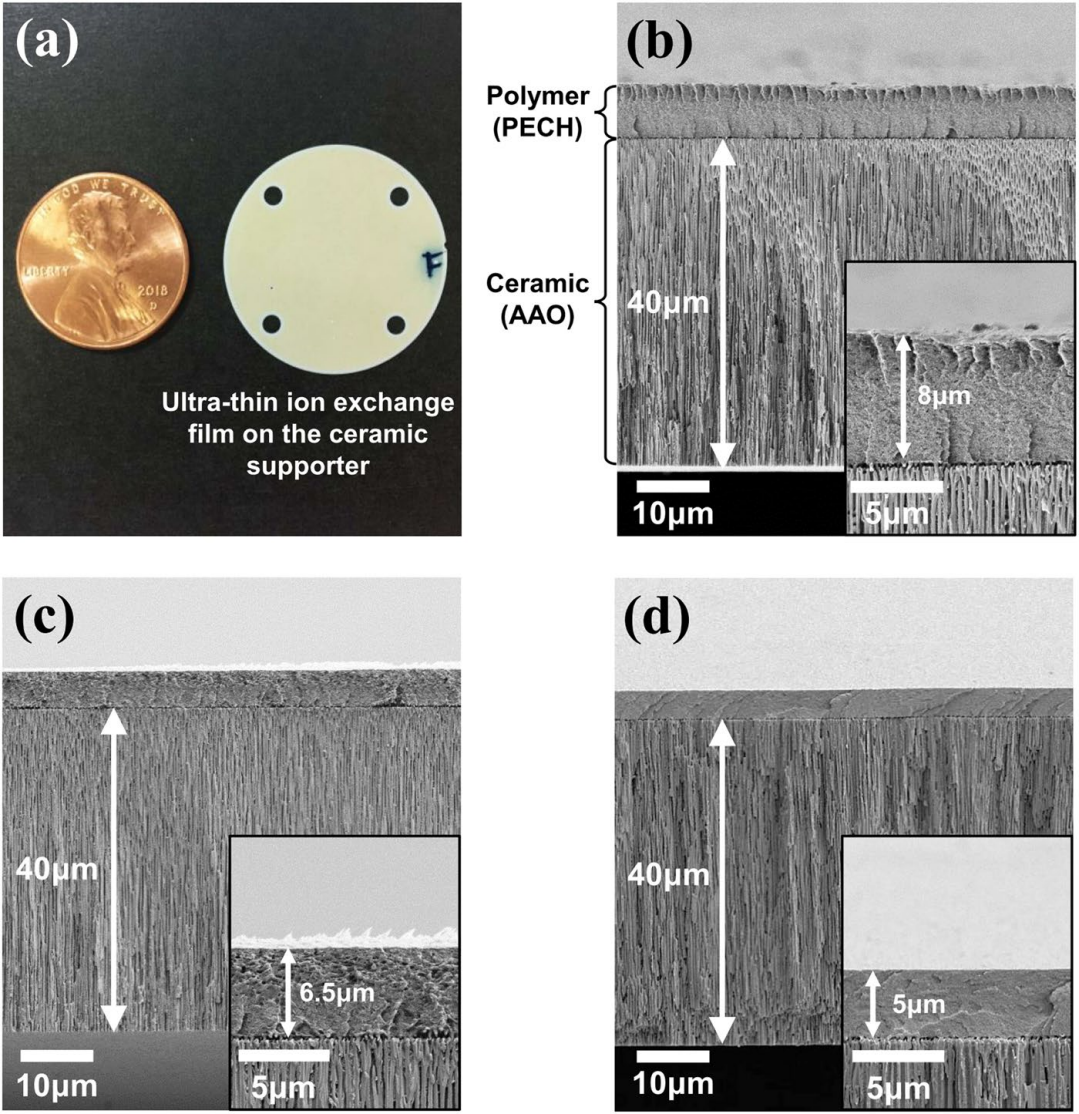
Images of fabricated ultra-thin ion exchange film on the ceramic supporter (UTFCS), (**a**) Real image of UTFCS, (**b**) scanning electron microscopy (SEM) images of 8 μm (polymer layer), (**c**) SEM images of 6.5 μm (polymer layer), (**d**) SEM images of 5 μm (polymer layer).

**Figure 6:**
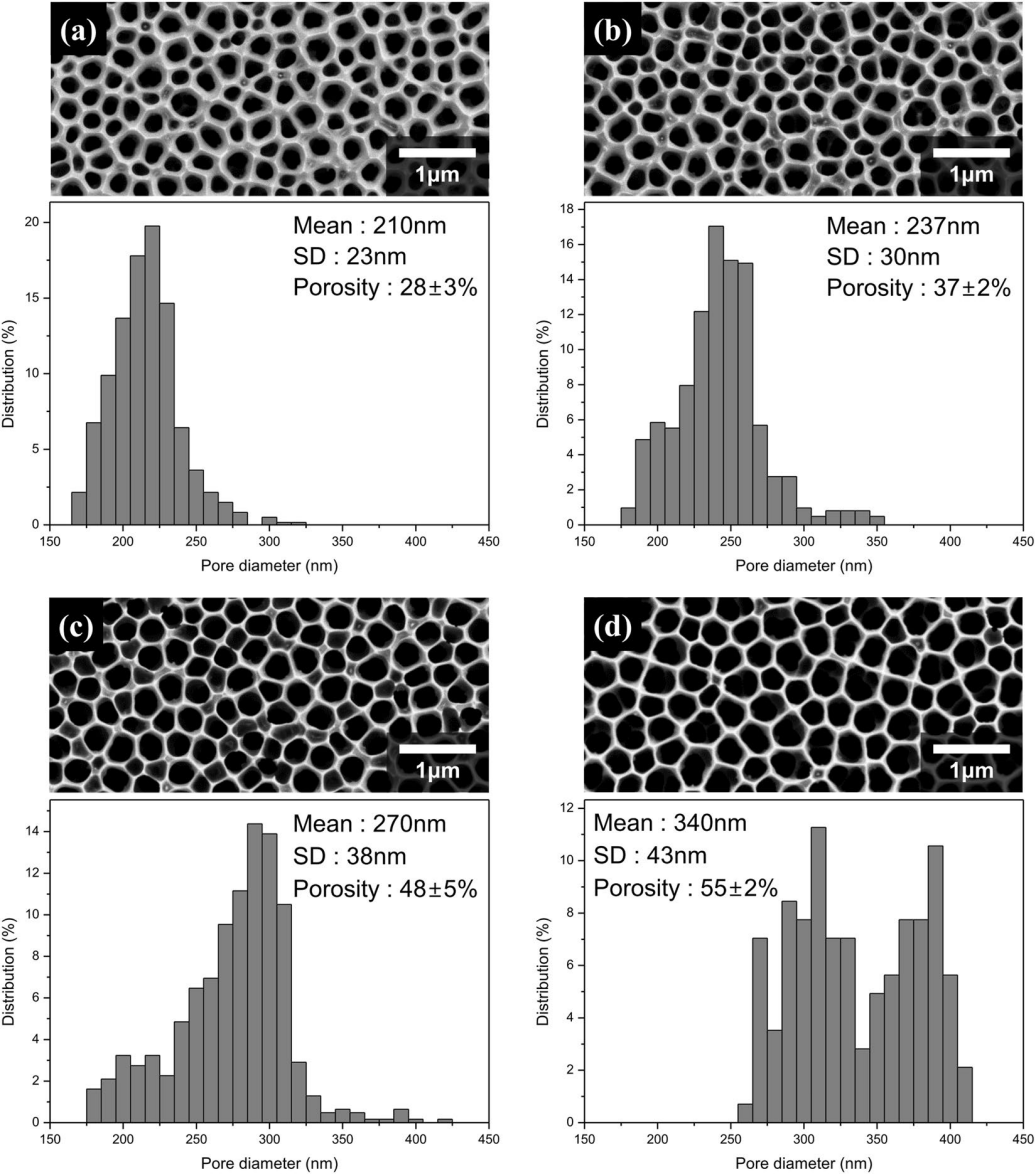
Scanning electron microscopy (SEM) images and porosity of nanoporous ceramic supporter, (**a**) 28%, (**b**) 37%, (**c**) 48%, (**d**) 55%.

**Figure 7:**
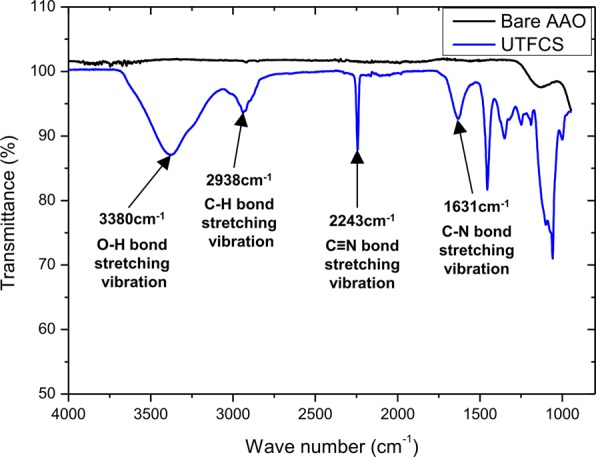
FT-IR spectrum of bare aluminum anodic oxide (AAO) and ultra-thin ion exchange film on the ceramic supporter (UTFCS).

UTFCSs were fabricated using the prepared nanoporous alumina and anion exchange membrane solution. The UTFCS was fabricated by spreading an anion exchange membrane solution using a ceramic supporter as the substrate, followed by spin coating. In order to investigate the effect of the polymer layer thickness on the electrical resistance in the UTFCS, the thickness of the polymer layer was controlled by controlling the spin coating speed. Figure 5 shows a real image and SEM images of the prepared UTFCS. The thicknesses of the polymer layers were 8 μm, 6.5 μm, and 5 μm, depending on the spin coating rate. In addition, to investigate the effect of the ceramic supporter porosity on the electrical resistance in the UTFCS, the porosity of the ceramic supporter was controlled by a pore widening process. Figure 6 shows the SEM images and porosity of the ceramic supporter according to the pore expansion process time. The pore expansion process was performed for 0, 1, 2, and 3 h and showed a 28%, 37%, 48%, and 55% porosity, respectively. Based on the inter-pore distance, the porosity could be up to 70%. However, because the ceramic supporter was used for mechanical stiffness, excessive pore expansion was not performed. Infrared spectroscopy (FT-IR) was performed to confirm the successful synthesis of the PECH polymer anion exchange membrane. PECH-based anion exchange membranes absorb infrared rays at 2927 cm^−1^ for the C-H bonds, 2240 cm^−1^ for the C≡N bonds, and 1640 cm^−1^ and 3380 cm^−1^ for the C-N and O-H bonds^21^. Figure 7 shows the FT-IR spectrum of the UTFCS. Absorption of the infrared rays occurred near the aforementioned wave number, and thus, synthesis of an anion exchange membrane was successfully accomplished.

### Characterization of the UTFCS

Electrical resistance was measured by a direct current method. Two chambers separated by an ion exchange membrane were filled with a 0.5 M NaCl solution. The current was applied to the platinum electrode, and the voltage formed across the membrane was measured by an Ag/AgCl reference electrode. The membrane potentials were generated when 0.5 M and 0.017 M NaCl solutions were filled in the two chambers separated by the ion exchange membrane and were measured using the Ag/AgCl reference electrode. 0.5 M and 0.017 M NaCl solutions were generally considered as usual seawater and river water concentration^2,29,30^. The permselectivity was calculated from the following equation^3^,

4 $${\rm{Permselectivity}}( \% )=\frac{{\psi }_{measured}}{{\psi }_{theoretical}}\times 100$$

where ${\psi }_{measured}$ is the measured membrane potential, and ${\psi }_{theoretical}$ is the theoretical membrane potential calculated by the Nernst equation.

### Configuration of the RED cell

**Figure 8:**
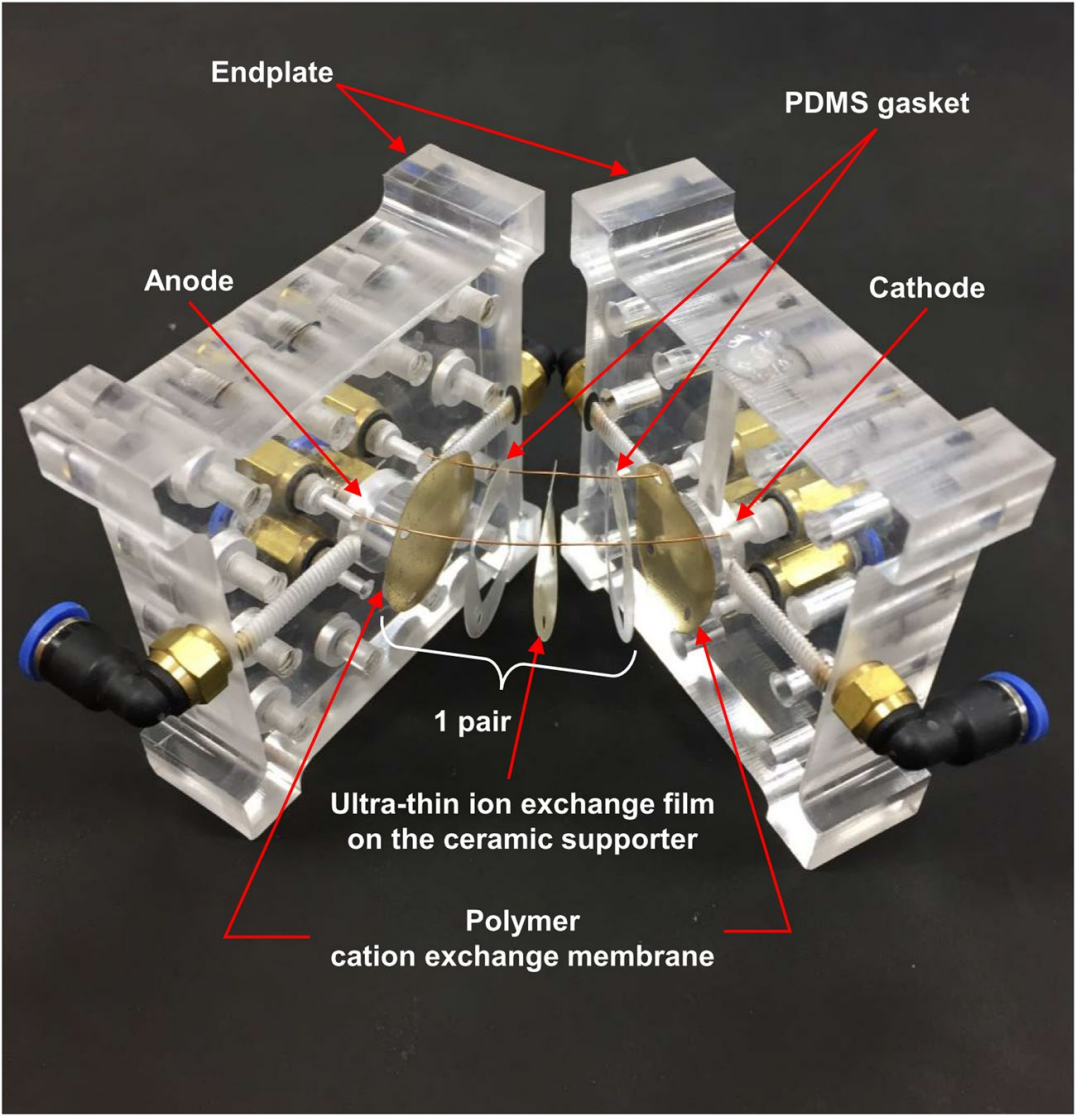
Real image of reverse electrodialysis (RED) cell. The inside of the cell was composed of a ultra-thin ion exchange film on the ceramic supporter (UTFCS), CMX cation exchange membrane, and PDMS gasket. Anode and cathode which were attached to the acrylic end plate at both ends of the cell.

Figure 8 shows the configuration of the RED cell. The inside of the cell was composed of a UTFCS, CMX cation exchange membrane, and PDMS gasket, and the anode and cathode were attached to the acrylic end plate at both ends of the cell. When injecting seawater and fresh water, it was bolted to prevent leakage.
